# Notes and News

**Published:** 1896-03-07

**Authors:** 


### Notes and News.

(Collected and Communicated.)

### HOSPITAL MEETINGS.

A General Court of Governors of the Royal Sea Bathing Infirmary, Margate, will be held on Saturday, March 7th, at 12.30 p.m., at the London offices, 30, Charing Cross, S.W.

The Annual Meeting of Governors of the National Society for Employment of Epileptics will be held at the offices, 12, Buckingham Street, Strand, on Monday, March 9th, at 5 p.m.

The Annual General Meeting of Governors of the St. Pancras and Northern Dispensary will be held at 126, Euston Road, on Tuesday, 10th inst., at 10.30 a.m.

The Annual General Meeting of Governors of the Dental Hospital, Leicester Square, will be held at the Hospital on Thursday, March 12th, at 3.30 p.m.

A Special Court of Governors of the Seamen's Hospital Society will be held in the Board-room, 24, Budge Row, Cannon Street, E.C., on Friday, March 13th, at 5.30 p.m.

The New Hospital for Women, 144, Euston Road, will hold its Annual General Meeting on March 10th at 3.30 p.m.

The Annual Court of Governors of the North London Hospital for Consumption will be held at the office, 41, Fitzroy Square, W., on Wednesday, March 18th, at 4 p.m.

---

The Queen has forwarded £10 towards the maintenance of the Royal National Hospital for Consumption at Ventnor.

---

We give on another page a report of several hospital meetings and dinners. They illustrate rather strikingly the bearings or the relative value of dinners and annual meetings which are energetically organised. The only hospital which attempts to get money at its annual meeting is the Seamen's Hospital, which has no dinner. It will be seen that the money announced at the Seamen's Hospital meeting was £1,605 net, that is, money obtained without any cost to the institution. This is a striking fact, which we commend to the consideration of hospital committees and secretaries. It reflects the greatest credit upon Mr. Michelli, the energetic secretary of the Seamen's Hospital, and redounds to the liberality and authority of the chairman, Sir Donald Currie, to whose efforts it is mainly due.

---

A dinner is an expensive matter, but in that given in connection with the Victoria Hospital for Children, Commander Blount introduced a novelty by charging 25s. a head to the guests, some 150 in number, who supported the Duke of York on Saturday, the 29th ult. If hospital dinners can be made so popular as to secure a payment of 25s. a head and a full attendance, as in the case of the Victoria Hospital, there will be little to be said against dinners on the ground of expense. The guests at Commander Blount's dinner had good value for their money, seeing that the speeches were far above the average. H.R.H. the Duke of York well held his own by comparison with his contemporaries who were present. Rear-Admiral Kennedy, Mr. H. F. Dickens, Q.C. (son of Charles Dickens), and Mr. Pickering Pick made admirable speeches, the former especially creating much amusement by his energy and wit. It was interesting to note that the younger speakers felt the stress of what was to follow the dinner, and that their conversational powers flagged until after their speeches were delivered. We congratulate the Victoria Hospital on the subscription of £3,000.

---

It was intimated a few weeks ago that Mr. Murdoch the chairman of the Great Northern Central Hospital, intended to correct his mis-statements in regard to the salaries paid to hospital secretaries and the cost per bed at various hospitals at the annual meeting on the 28th ult. Much surprise and some indignation has been caused by the chairman's silence on both points, and it is plainly stated that there is no other gentleman occupying a similar position in the hospital world who would have failed to avail himself of the first opportunity of correcting the errors in question. We are informed that several private remonstrances have been addressed to the chairman, and, in reply to inquiries, we have to state that we shall be glad to publish any correction of the statements in question which may be sent us by the authorities of the institutions who feel themselves aggrieved.

---

The scene at Brighton on Saturday, when the Prince of Wales laid the foundation-stone of the new out-patients' department at the Sussex County Hospital, was a very brilliant one. Great preparations had been made for the Royal visit, and the town was gay with flags, banners, and mottoes. The ceremony took place in a spacious marquee in front of the hospital. The Prince, escorted by a large military escort, arrived at one o'clock. He was accompanied by the Duke and Duchess of Fife and Major-General Stanley Clarke. After the usual addresses had been delivered and the stone duly laid, the Duchess of Fife came forward and received purses of money, presented by lady collectors. In this manner £1,500 has been added to the building fund. The ceremony concluded by an address of thanks to H.R.H. the Prince of Wales, delivered by the Duke of Norfolk.

---

The thanks of the governors of the Great Northern Central Hospital are due to Mr. R. H. Bax for calling attention at the annual meeting to the fact that the cost of printing, stationery, postage, and appeals amounted to £831. He pertinently asked if this sum included "the cost of distributing the *Charity Record*, containing a report of the meeting on December 30th last, how much was paid for such copies, and what was the total sum received by the proprietors of this print from the hospital during the year?" The Chairman replied that it had been the custom to send out this print to the governors for many years past, and he thought such expenditure "was only a slight return for the benefits received from the publication in question." He gave no idea of the amount paid, however. It seems incredible, but it is undoubtedly true, that instead of saving the hospital expenditure by employing a reporter and circulating their own report of the proceedings, the committee of the Great Northern Hospital should continue a practice which is scouted by every reputable institution, not only on the grounds of economy, but of self-respect and good management. Anyone who wants to know the abuses which may attach to the practice to which Mr. Bax called attention should read an article, "Hospital Promotion—A Wag's Experience," in The Hospital for November 20th, 1886, p. 122.

